# Severe COVID-19 by SARS-CoV-2 Lineage B.1.1.7 in Vaccinated Solid-Organ Transplant Recipients: New Preventive Strategies Needed to Protect Immunocompromised Patients

**DOI:** 10.3390/vaccines9080806

**Published:** 2021-07-21

**Authors:** Daniela Loconsole, Emma Diletta Stea, Anna Sallustio, Giulia Fontò, Virginia Pronzo, Simona Simone, Francesca Centrone, Marisa Accogli, Loreto Gesualdo, Maria Chironna

**Affiliations:** 1 Department of Biomedical Sciences and Human Oncology-Hygiene Section, University of Bari, 70124 Bari, Italy; daniela.loconsole@uniba.it (D.L.); francesca.centrone.fc@gmail.com (F.C.); accoisa@gmail.com (M.A.); 2 Nephrology, Dialysis and Transplantation Unit, Department of Emergency and Organ Transplantation, University of Bari, 70124 Bari, Italy; emmadiletta.stea@libero.it (E.D.S.); giuliafonto@gmail.com (G.F.); virginia.pronzo@gmail.com (V.P.); simonasimone1976@gmail.com (S.S.); loreto.gesualdo@uniba.it (L.G.); 3 Azienda Ospedaliero-Universitaria Consorziale Policlinico di Bari, Hygiene Unit, 70124 Bari, Italy; annasallustio@libero.it

**Keywords:** solid-organ transplant recipients, SARS-CoV-2 infection, B.1.1.7 lineage, COVID-19 vaccine, whole-genome sequencing

## Abstract

Background: Solid-organ transplant (SOT) recipients are at a high risk of severe COVID-19, and are priority for vaccination. Here, we describe three cases of severe COVID-19 caused by SARS-CoV-2 B.1.1.7 lineage in vaccinated SOT recipients. Methods: Three SOT patients were hospitalized in the Policlinico Hospital of Bari (southern Italy) and underwent nasopharyngeal swabs for molecular detection of SARS-CoV-2 genes and spike protein mutations by real-time PCR. One sample was subjected to whole-genome sequencing. Results: One patient was a heart transplant recipient and two were kidney transplant recipients. All were hospitalized with severe COVID-19 between March and May 2021. Two patients were fully vaccinated and one had received only one dose of the BNT162b2 mRNA vaccine. All the patients showed a high viral load at diagnosis, and molecular typing revealed the presence of B.1.1.7 lineage SARS-CoV-2. In all three cases, prolonged viral shedding was reported. Conclusions: The three cases pose concern about the role of the B.1.1.7 lineage in severe COVID-19 and about the efficacy of COVID-19 vaccination in immunocompromised patients. Protecting immunocompromised patients from COVID-19 is a challenge. SOT recipients show a suboptimal response to standard vaccination, and thus, an additive booster or a combined vaccination strategy with mRNA, protein/subunit, and vector-based vaccines may be necessary. This population should continue to practice strict COVID-19 precautions post-vaccination, until new strategies for protection are available.

## 1. Introduction

COVID-19 is a major concern in solid-organ transplant (SOT) recipients, which have a mortality rate of up to 20% [1,2], and a 30% increased risk of death or use of mechanical ventilation [3]. In particular, heart transplant recipients have a more severe clinical course compared with the general population [4] and an estimated fatality rate of 25% [5]. Although they seem to not be at a higher risk of transmission [4]. Some studies demonstrated that SOT recipients hospitalized with COVID-19 showed similar overall outcomes as hospitalized non-SOT recipients with other chronic conditions (obesity, diabetes, and hypertension), suggesting that chronic immunosuppression may not be an independent risk factor for poor outcomes in COVID-19 patients requiring hospitalization [6]. However, other studies reported that SOT status independently increases the risk of hospital admission and acute kidney injury (AKI) in COVID-19 patients [7]. Bajpai et al. reported a 29% rate of post-COVID-19 AKI in kidney transplant recipients [8].

SOT recipients have been considered among the prioritized groups for COVID-19 vaccination since the beginning of the immunization campaign with the BNT162b2 mRNA vaccine in December 2020 [9]. Transplant recipients were excluded from COVID-19 vaccine clinical trials, and since the immune response elicited in this population could be weaker than in healthy individuals [10], the efficacy, durability, and safety of COVID-19 vaccines in immunocompromised patients are yet to be established [11]. Recent studies showed evidence of an impaired humoral response to the BNT162b2 mRNA vaccine in SOT recipients [12,13]. In particular, Korth et al. demonstrated that the humoral response of renal transplant recipients after two doses of the BNT162b2 mRNA COVID-19 vaccine was significantly lower than that of healthy controls [14]. The current data suggest that SOT recipients might be vulnerable to COVID-19 despite their vaccination status [14]. The data regarding immune-suppressed patients are probably explained by the lack of competent host immunity, which is needed to generate a fully protective response after immunization [15]. However, the nature and extent of immunosuppression in SOT patients is still debated and further information is needed on this topic.

Here, we report three cases of severe COVID-19 caused by SARS-CoV-2 B.1.1.7 lineage in vaccinated SOT recipients.

## 2. Patients and Methods

Three SOT patients were hospitalized in the sub-intensive Nephrology, Dialysis, and Transplantation Unit of the Policlinico Hospital of Bari (southern Italy) for COVID-19 between 12 March 2021 and 07 May 2021. One patient was a heart transplant recipient and two were kidney transplant recipients. All three patients received conventional maintenance immunosuppressive therapy, which includes a calcineurin inhibitor in combination with an antimetabolite (mycophenolate mofetil) and a low dose of steroids. The clinical presentation was classified according to the National Institute of Health (NIH) clinical staging of COVID-19 disease [16]. Nasopharyngeal swabs (NPS) (FLOQSwabs, Copan Italia, Brescia, Italy) were collected on the day of hospital admission and were analyzed in the Laboratory of Molecular Epidemiology and Public Health of the Hygiene Unit (Policlinico Hospital of Bari), which is the coordinator of the Regional Laboratory Network for SARS-CoV-2 diagnosis. The molecular test was performed using a three-target (N, ORF1ab, and S genes) commercial multiplex real-time PCR assay from Thermo Fisher Scientific (TaqPath RT-PCR COVID-19 Assay). Samples were also screened for the presence of notable of spike protein mutations (69–70 deletion, N501Y, K417N, E484K, and K417T) using a commercial multiplex real-time PCR kit (Seegene Allplex SARS-CoV-2 Variants I Assay, Arrows Diagnostics, Genova, Italy). Only the sample collected from the heart transplant recipient was available for molecular characterization. Whole-genome sequencing was performed using the Ion Torrent platform (Thermo Fisher Scientific, Waltham, MA, USA) as previously described [17].

Serum samples were collected from the three patients during hospitalization. For the heart transplant recipient, the Elecsys Anti-SARS-CoV-2 Assay on the Cobas e801 was performed according to the manufacturer’s instructions (Roche Diagnostics, Monza, Italy). This electro-chemiluminescence assay (ECLIA) is based on a modified double-antigen sandwich immunoassay using recombinant N protein and is used for the specific detection of total SARS-CoV-2 antibodies, including IgM and IgG. The results were reported as negative if <1.0 AU/mL and positive if ≥1.0 AU/mL. For the other two SOT recipients, an ECLIA that detects IgG against the receptor-binding domain (RBD) of the spike protein of SARS-CoV-2 was performed (Elecsys Anti-SARS-CoV-2 S Assay, Roche Diagnostics, Monza, Italy). A value of <0.80 BAU/mL was considered non-reactive and a value of ≥0.80 BAU/mL was considered reactive, according to the manufacturer’s instructions. For the three patients, two different types of antibody tests were performed since the ECLIA assay that detects IgG against the RBD of the spike protein was not available during the hospitalization of the heart transplant recipient. IgG anti-RBD antibodies were also detected in 14 serum samples collected from healthy healthcare workers without SARS-CoV-2 infection after 7 and 28 days from the second dose of the BNT162b2 mRNA vaccine. These samples were considered as control samples for the study. The geometric mean titres (GMTs) for SARS-CoV-2 spike-specific IgG were reported.

Ethical review and approval were waived for this study since all activities were conducted as part of the legislated mandate of the Health Promotion and Public Health Department of Apulia. All procedures were carried out in accordance with the guidelines for research on human subjects of the Declaration of Helsinki, as revised in 2013. Informed written consent for publication was obtained from all subjects involved in the study.

## 3. Results

Patient n.1 was a 65-year-old male who had undergone a heart transplant in 2014 because of a primitive dilated cardiomyopathy (Table 1). He was fully vaccinated with the BNT162b2 COVID-19 mRNA vaccine, having received two doses in accordance with the recommended schedule. Eighteen days after the second dose, he developed diarrhoea, vomiting, and dyspnoea. The NPS tested positive for SARS-CoV-2 infection by real-time PCR. The patient had shown severe COVID-19 since the onset of symptoms, requiring home oxygen therapy. He was hospitalized with severe pneumonia on 17 March 2021. The number of white blood cells (WBCs) at the admission was 6.32 × 10^3^/μL and the percentage of lymphocytes was 25.1%. During hospitalization, the patient was treated with i.v. dexamethasone (6 mg once daily for 10 days, followed by a fast taper) and s.c. enoxaparin (4000 UI daily for 1 month). Piperacillin tazobactam was added in agreement with an infectious disease specialist to prevent bacterial co-infection. The anti-proliferative agent (mycophenolate mofetil) was interrupted and the calcineurin inhibitor (cyclosporine) dose was reduced. Continuous positive airway pressure (CPAP) was required for 2 weeks, followed by high flow nasal cannula (HFNC) in order to improve oxygenation, unload respiratory muscles, and avoid intubation. He completely recovered and was discharged on 8 April 2021.

Patient n.2 was a 48-year-old female who received a kidney transplant in 2015 because of end-stage renal disease of unknown origin. The patient was fully vaccinated with the BNT162b2 COVID-19 mRNA vaccine, having received the second dose on 20 January 2021. On 1 April 2021, she developed a dry cough, dyspnea, and fever. Suspecting a bacterial infection, empirical antibiotic therapy (ciprofloxacin 500 mg daily) was administered for 1 week, coupled with home oxygen therapy. As clinical symptoms persisted despite the antibiotics, COVID-19 was suspected and confirmed by a positive PCR test from a NPS (11 April 2021). She was hospitalized with pneumonia and treated with i.v. dexamethasone (4 mg once daily for 4 days, followed by a fast taper), s.c. enoxaparin (4000 UI daily), and oxygen therapy with a low-flow nasal cannula. The number of WBCs at the admission was 4.42 × 10^3^/μL and the percentage of lymphocytes was 12.0%. The mycophenolate mofetil was withdrawn and tacrolimus was maintained. Due to her favourable clinical and radiologic condition, she was discharged after 4 days.

Case n.3 was a 52-year-old male affected by IgA nephropathy who received a kidney transplant in 2005. He presented with high-grade fever and dyspnoea on 8 April 2021 and tested positive for SARS-CoV-2 infection on the same day. This patient also received oxygen therapy at home, but was admitted to the hospital on 22 April 2021 with a pulmonary embolism. During the hospitalization, he was treated with s.c. enoxaparin (6000 UI twice a day for 2 weeks) and i.v. dexamethasone (6 mg once daily for 7 days, followed by a fast taper). The number of WBCs at the admission was 10.63 × 10^3^/μL and the percentage of lymphocytes was 4.6%. The mycophenolic acid and the cyclosporine were withdrawn. Non-invasive bi-level ventilation followed by HFNC were required to improve the oxygenation. He was discharged on 7 May 2021. Only one dose of the BNT162b2 COVID-19 mRNA vaccine had been administered to this patient, on 27 March 2021, 12 days before clinical onset.

The immunosuppressive therapy was modified according to the Italian Consensus on Kidney Transplant Recipients [18] for all three patients.

Real-time PCR for SARS-CoV-2 on samples from the three cases showed cycle threshold (Ct) values <20 and S-gene target failure (SGTF), which is considered a robust proxy for the SARS-CoV-2 B.1.1.7 lineage [19]. In all three cases, prolonged viral shedding was reported (Table 1). The samples were then subjected to molecular screening, which identified the B.1.1.7 variant because of the presence of the 69–70 deletion and the N501Y spike mutation. WGS performed on the sample collected from patient n.1 confirmed that the strain belonged to the Pangolin lineage B.1.1.7 (GISAID accession number: EPI_ISL_2365924).

Epidemiological investigations revealed that the source of the SARS-CoV-2 infection in the three SOT recipients were members of their households. Secondary cases were reported for patients n. 1 and n. 3.

Antibody tests were performed during hospitalization. For the heart transplant recipient, anti-SARS-CoV-2 IgG was detected with the following values: 11.6 AU/mL on 24 March 2021, 14.44 AU/mL on 27 March 2021, and 23.01 AU/mL on 6 April 2021. For the other two patients, anti-spike SARS-CoV-2 IgG was detected with the following values: 261 BAU/mL on 14 April 2021 for patient n.2 and 158 BAU/mL on 27 April 2021 for patient n.3. For the control samples, the GMTs for SARS-CoV-2 spike-specific immunoglobulin G were 2539.4 BAU/mL and 1859.8 BAU/mL after 7 and 28 days from the second dose of the BNT162b2 mRNA vaccine, respectively.

## 4. Discussion

In the present study, three cases of severe COVID-19 caused by SARS-CoV-2 B.1.1.7 lineage in vaccinated SOT recipients are reported. These cases pose concern about the role of the B.1.1.7 lineage in causing severe COVID-19 and about the efficacy of COVID-19 vaccination in immunocompromised patients.

It has been demonstrated that subjects infected by SARS-CoV-2 VOC 202012/01 are at a higher risk of severe COVID-19, hospitalization, and death [19,20]. Therefore, the severity of COVID-19 in the SOT recipients, as described here, could be related to infection with the B.1.1.7 variant.

Prolonged shedding of viable SARS-CoV-2 has been demonstrated in kidney transplant recipients as a result of a weak immunological pressure, which is also thought to allow the possible emergence of SARS-CoV-2 variants [21]. Prolonged viral shedding was detected in the cases described here. Although we did not perform viral culture to evaluate the presence of viable SARS-CoV-2 [21]. At the diagnosis stage, the samples showed low Ct values, which can be considered an indirect index of viral load [22]. This could explain the generation of secondary cases among the close contacts of two patients. The high viral load detected in samples from the three patients with severe COVID-19 could be also related to the presence of the B.1.1.7 variant [23,24]. Contact tracing revealed that all the three SARS-CoV-2 infections were acquired from the households of the patients. To contain a further diffusion of the infection among family members after the hospital discharge, non-pharmaceutical interventions, such as the isolation of cases, were adopted until the negative molecular test for SARS-CoV-2.

The three severe COVID-19 cases in SOT recipients are worrisome in light of the role of COVID-19 vaccination in protecting immunocompromised patients. A recent study by Ali et al. reported 14 cases of COVID-19 in fully vaccinated SOT recipients, 50% of whom required hospitalization and one of whom died [25]. In a recent study on SOT recipients, 54% of patients showed detectable IgG anti-SARS-CoV-2 antibodies after two doses of COVID-19 mRNA vaccine [12]. Studies have reported a humoral response in 49% of heart transplant recipients [15] and 22–36.4% of renal transplant recipients vaccinated with two doses of BNT162b2 mRNA vaccine [14,26]. Moreover, older heart transplant recipients and patients using antimetabolite immunosuppression were more likely to show low immunogenicity [12,15]. However, lowering the immunosuppressive regimen to enhance the vaccine response is not recommended due to the high risk of rejection [27]. In the cases described in the present study, the low humoral response could also be related to the use of antimetabolite immunosuppression since the drug was interrupted only at the COVID-19 onset. It is known that the detectable humoral response after two vaccine doses in SOT recipients is poor [12]. The current data suggest that a substantial proportion of transplant recipients still remain at risk of SARS-CoV-2 infection in spite of their vaccination status [12,13]. In the cases described here, we can speculate that the antibody response after vaccination was poor, since the antibody titers after the infection were much lower than those detected in the vaccinated control samples. However, we cannot compare the antibody titers with reference values since no immunological correlate of protection has currently been identified for COVID-19 vaccines.

Cases of SARS-CoV-2 infection have been reported also in vaccinated healthy subjects with anti-spike IgG, even though with mild symptoms [24]. In the cases of three SOT recipients here described, despite the severity of the illness, all patients recovered, suggesting a possible partial protection provided by the vaccine.

In a recent study, almost 40% of transplant recipients who seemed to not respond after the first COVID-19 vaccine dose demonstrated seroconversion after the second dose [15]. Thus, a possible strategy based on an additive booster vaccine in immunocompromised patients may help to enhance immune responses in this population. A recent report showed that the administration of a third dose of COVID-19 vaccine in SOT recipients led to an increase in antibody titers in one-third of patients with a negative antibody test after the second dose and in all patients who had low antibody titers after the second dose [28]. Moreover, in line with the influenza vaccine strategy [29], a vaccine with a higher antigen dose may result in stronger immune response in immunocompromised patients [15]. At the time of writing, for immunocompromised patients, COVID-19 mRNA vaccines are recommended. However, it might be possible that different types of vaccines, such as vector-based vaccines, could induce a better humoral response [14].

The nature and extent of immunosuppression in SOT patients is still controversial and unclear. Recent studies demonstrated that, in the general population, RBD antibodies from memory B cells persist despite a decrease in the antibody response to RBD [30,31]. A SARS-CoV-2-specific T cell response has also been demonstrated after infection in immunocompromised patients [32], suggesting that the cellular response to the COVID-19 vaccine may also be crucial in protecting this population from SARS-CoV-2 infection. In immunocompromised patients, the cellular immune response is also likely impaired, as two of three patients described in this study were fully vaccinated.

This study has some limitations. First, only one sample was available for WGS. However, the molecular screening for variants showed the presence of SARS-CoV-2 B.1.1.7 lineage, which was the prevalent strain circulating in Italy [33]. Second, serum samples collected after vaccination and before symptom onset for anti-spike IgG detection were not available. Nevertheless, the low antibody titers detected after infection may reflect the humoral response to vaccination.

Further studies are needed to assess a more efficient individualized COVID-19 vaccination strategy in immunocompromised patients that may include more than two booster doses or a combined mRNA, protein/subunit, and vector-based vaccination strategy [14]. Moreover, promoting the vaccination of close household contacts of SOT recipients, so-called “ring vaccination”, could also reduce the chance of direct household spread of SARS-CoV-2 infection [34]. This population should continue to practice strict COVID-19 precautions post-vaccination, particularly in light of the worldwide worrisome spread of SARS-CoV-2 variants of concern (VOCs), such as the Delta VOC (B.1.617.2 lineage). SARS-CoV-2 Delta VOC represents a global concern since an increased transmissibility, a potential reduction in neutralization by some monoclonal antibody treatments, and a potential reduction in neutralization by post-vaccination sera have been demonstrated [35].

Protecting immunocompromised patients is a challenge for the future and new strategies for protection are needed to ensure better immune responses. Continuous monitoring of SARS-CoV-2 infection in these at-risk patients is advisable at this point of the immunization campaign.

## Figures and Tables

**Table 1:** Demographic and clinical characteristics of the three solid-organ transplant recipients with COVID-19.

Patient Number	Solid Organ Transplant	Age	Sex	Comorbidity	Onset of Symptoms	Clinical Presentation	CT Scan	Length of Hospital Stay (Days)	Days from Symptom Onset and First Negative NPS	Vaccinated
1	Heart	65	Male	Hypertension, type 2 diabetes, HCV-related hepatic disease	12 March 2021	Dyspnoea, diarrhoea, vomiting	Multiple ground-glass opacities	23	35	Yes (BNT162b2 COVID-19 mRNA vaccine, dose 2 on 23 February 2021)
2	Kidney	48	Female	Hypertension	1 April 2021	Dry cough, dyspnoea	Ground-glass opacity	4	25	Yes (BNT162b2 COVID-19 mRNA vaccine, dose 2 on 20 January 2021)
3	Kidney	52	Male	Hypertension, hypertensive heart disease	8 April 2021	High-grade fever, dyspnoea	Pulmonary embolism	16	28	Yes (BNT162b2 COVID-19 mRNA vaccine, dose 1 on 27 March 2021)

CT = computed tomography; NPS = nasopharyngeal swab.

## Data Availability

All data regarding the patients and laboratory tests are available from the corresponding author by e-mail request.
